# Neuroprotective Effect of Tricyclic Pyridine Alkaloids from *Fusarium lateritium* SSF2, against Glutamate-Induced Oxidative Stress and Apoptosis in the HT22 Hippocampal Neuronal Cell Line

**DOI:** 10.3390/antiox9111115

**Published:** 2020-11-11

**Authors:** Dahae Lee, Hyun Gyu Choi, Ji Hye Hwang, Sang Hee Shim, Ki Sung Kang

**Affiliations:** 1 College of Korean Medicine, Gachon University, Seongnam 13120, Korea; pjsldh@gachon.ac.kr (D.L.); jhhani@gachon.ac.kr (J.H.H.); 2 College of Pharmacy, Duksung Women’s University, Seoul 01369, Korea; chg---@hanmail.net

**Keywords:** glutamate, oxidative stress, Ca^2+^, apoptosis, HT22 cells, *Fusarium lateritium* SSF2

## Abstract

Excessive glutamate damages neuronal cells via the accumulation of intracellular reactive oxygen species (ROS), calcium ion (Ca^2+^) influx, depolarization of mitochondrial membrane potential, and apoptosis, which may result in the development of chronic neurodegenerative diseases. In this study, we evaluated the effects of 4,6′-anhydrooxysporidinone isolated from endophytic fungus *Fusarium lateritium* SSF2 on glutamate-induced cytotoxicity, accumulation of intracellular ROS, increases in superoxide anion production, Ca^2+^, depolarization of mitochondrial membrane potential, and apoptotic cell death in hippocampal HT22 cells. 2′,7′-Dichlorofluorescin diacetate (H2DCFDA) staining was used to determine the intracellular reactive oxygen species concentration and dihydroethidine (DHE) staining was used to determine the superoxide radical. Expression of the nuclear factor-erythroid-2–related factor 2 (Nrf2) and heme oxygenase-1 (HO-1) was analyzed by Western blot. Fluo-4 staining was used to determine the intracellular Ca^2+^ levels. In order to explore mitochondrial membrane potential, tetramethylrhodamine methyl ester (TMRM) staining was used. Apoptotic cell death was evaluated using Annexin-V/propidium iodide (PI) staining and TUNEL staining. Expression of the cytochrome *c* release and cleaved caspase-9, -3 was analyzed by Western blot. Here, we were able to isolate 4,6′-anhydrooxysporidinone from endophytic fungus, *Fusarium lateritium* SSF2, which was shown to protect HT22 cells from glutamate-induced cytotoxicity, accumulation of intracellular ROS, increases in superoxide anion production, Ca^2+^, and depolarization of mitochondrial membrane potential. In addition, 4,6′-anhydrooxysporidinone enhanced the expressions of Nrf2 and HO-1. It also inhibited the apoptotic cell death through the inhibition of cytochrome *c* release and cleaved caspase-9, -3 in glutamate-treated HT22 cells. Therefore, our results provide ample evidence of the neuroprotective properties of 4,6′-anhydrooxysporidinone.

## 1. Introduction 

The progression of various neurological diseases, such as Alzheimer’s disease (AD), Parkinson’s disease and ischemic brain injuries may be the result of neuronal cell death in the central nervous system (CNS) [1]. Although glutamate plays several important roles within the CNS, acting as an excitatory neurotransmitter [2], neuronal cell death may be caused by exposure to high concentrations of glutamate. In the neurodegenerative disease processes, mitochondrial Ca^2+^ homeostasis and imbalance between antioxidant defenses and reactive oxygen species (ROS) is critical to prevent neuronal cell death [3]. High levels of glutamate induce elevated intracellular Ca^2+^ levels by membrane depolarization [4]. In addition, elevated intracellular Ca^2+^ levels are responsible for the opening of the mitochondrial permeability transition pore and apoptotic cell damage [5]. In addition, destruction of mitochondrial homeostasis causes overproduction of ROS and increases release of cytochrome *c*, which has a role in regulating the mitochondrial-mediated apoptosis pathway [6]. Nuclear factor-erythroid-2–related factor 2 (Nrf2), as a regulator of cellular antioxidant function, plays an important role in the functional integrity of the mitochondria [7].

Both glutamate antagonists and various natural antioxidant compounds have been widely studied for their application in neuroprotection [8]. It has also been reported that cyanidin-3-glucoside, an anthocyanin derived from berries, acts as an Nrf2 inducer. It also reduces oxidative stress, endoplasmic reticulum stress, and apoptosis in glutamate-treated HT22 cells [9]. Another recent study demonstrated the protective effect of artemisinin, a sesquiterpene lactone, on glutamate-induced HT22 cell death, with this compound also attenuating oxidative stress and apoptosis [10]. Recent evidence suggests that the neuroprotective effect of obovatol, a neolignan isolated from *Magnoliae Cortex*, is effected by a decrease in intracellular ROS generation leading to mitogen-activated protein kinase (MAPK) phosphorylation [11]. Another study suggested that kaempferol, a flavonol isolated from *Petasites japonicus*, exerts a protective effect against glutamate-induced HT22 cell death. It has also been shown that kaempferol inhibits oxidative stress and calcium influx [12]. These studies suggest that the administration of antioxidant compounds could help to prevent neurological disease by protecting hippocampal cells from the oxidative damage induced by high concentrations of glutamate.

However, relatively few studies have focused on the antioxidant and neuroprotective effects of fungi and their active compounds, despite their acknowledged contribution to the field of natural products and their demonstrated effect on glutamate-induced neuronal cell death. Adenosine isolated from *Cordyceps cicadae*, a parasitic fungus that grows mainly on the larvae of Cicada flammata Dist., protects the neuroendocrine cell line PC12 from glutamate-induced cytotoxicity, intracellular ROS accumulation, Ca^2+^ influx, and apoptosis [13]. In addition, daidzein-7-α-l-rhamnopyranoside was isolated from fungus-growing termite-associated *Streptomyces* sp., while RB1 inhibits glutamate-induced cell death in HT22 cells [14]. In N18-RE-105 neuroblastoma-retina hybrid cells, butenolide derivatives isolated from *Aspergillus terreus* significantly improved glutamate-induced excitotoxicity and exhibited radical scavenging activities [15]. These studies demonstrate that fungi produce a variety of neuroprotective compounds, suggesting that their further investigation may uncover more potential therapeutics in the future.

Protective effects of tricyclic pyridone alkaloids isolated from endophytic fungus, *Fusarium lateritium* SSF2 against glutamate-induced HT22 cell death have not been studied before. Therefore, this study focused on the effects of compounds against glutamate-induced cytotoxicity, accumulation of intracellular ROS, increases in superoxide anion production and Ca^2+^, depolarization of mitochondrial membrane potential, and apoptotic cell death, and serves as evidence that 4,6′-anhydrooxysporidinone from *F. lateritium* exerts a protective effect against glutamate-mediated neuronal cell death in HT22 cells.

## 2. Materials and Methods 

### 2.1. Extraction and Isolation 

Fungal strain SSF2 was cultured on PDA media and these media were then extracted with methanol three times to create the MeOH extracts (1.78 g). The methanol extracts were suspended in 500 mL of distilled water, fractionated in the order n-hexane, CHCl_3_, and EtOAc, and then concentrated under reduced pressure to obtain the respective solvent fractions. The CHCl_3_ fraction was subjected to open column chromatography over SiO_2_ with a gradient elution of n-hexane-acetone (0–100%) to obtain eight fractions (FA-FH). Fraction FE (32.4 mg) was subjected to semi-preparative HPLC (Lunar 5 µm, C18, 250 × 10 mm) with an elution in CH_3_CN/H_2_O (70:30 to 100:0) to purify compound **1** (18.7 mg). Fraction FF (29.0 mg) was also subjected to semi-preparative HPLC (Lunar 5 µm, C18, 250 × 10 mm) with an elution in CH_3_CN/H_2_O (70:30 to 100:0) to produce compounds **2** (7.7 mg) and **3** (2.7 mg). 

### 2.2. Cell Culture

HT22 mouse hippocampal cells (purchased from Korean Cell Line Bank (Seoul, Korea)) were grown to 80% confluence in Dulbecco’s modified Eagle’s medium (DMEM; Cellgro, Manassas, VA, USA) supplemented with 1% penicillin/streptomycin (Invitrogen Co., Grand Island, NY, USA) and 10% fetal bovine serum (Atlas, Fort Collins, CO, USA) in a humidified atmosphere at 37 °C supplemented with 5% CO_2_.

### 2.3. Measurement of Cell Viability

HT22 cells were grown for 24 h and then treated with 5 mM glutamate (Sigma, St. Louis, MO, USA) with or without compounds 1-3 and N-acetylcysteine (NAC), and then incubated for a further 24 h. After treatment, 10 µL of Ez-CyTox reagent (Daeil Lab Service, Seoul, Korea) was added to each well containing adherent and non-adherent cells in accordance with the manufacturer’s instructions. After incubation for 30 min, absorbance at 450 nm was measured using an E-Max microplate reader (Molecular Devices, Sunnyvale, CA, USA) [16]. 

### 2.4. Measurement of Intracellular Reactive Oxygen Species

Cells were treated with 5 mM glutamate with or without SSF2-2 and NAC for 8 h, and then 10 µM 2′,7′-dichlorofluorescin diacetate (DCFDA; Sigma, St. Louis, MO, USA), an indicator of ROS [17], was added to each well containing adherent and non-adherent cells and incubated for another 30 min. The HT22 cells were then washed three times in phosphate-buffered saline (PBS) and the fluorescent intensity of DCF was measured at 495/517 nm (*ex*/*em*) using a SPARK 10M fluorescent microplate reader (Tecan, Männedorf, Switzerland). Fluorescent images were taken using an IX50 fluorescent microscope equipped with a CCD camera (Olympus, Tokyo, Japan).

### 2.5. Measurement of Intracellular Ca^2+^

Cells were treated with 5 mM glutamate with or without SSF2-2 and NAC for 8 h and then 2 µM Fluo-4 AM (Invitrogen, Eugene, OR, USA), a membrane-permeable fluorescent indicator for Ca^2+^, was added to each well (1 × 10^4^ cells per well) containing adherent and non-adherent cells and incubated for another 30 min. These cells were then washed with serum and phenol red-free DMEM medium three times. The fluorescent intensity of Fluo-4 AM was quantified using ImageJ software (National Institutes of Health, Bethesda, MD, USA). Fluorescent images were taken using an IX50 fluorescent microscope equipped with a CCD camera (Olympus, Tokyo, Japan).

### 2.6. Measurement of Superoxide Radical

Cells were treated with 5 mM glutamate with or without SSF2-2 and NAC for 8 h and then 50 μM dihydroethidine (DHE; Cayman Chemical, Ann Arbor, MI, USA), an indicator of superoxide, was added to each well (1 × 10^4^ cells per well) containing adherent and non-adherent cells and incubated for another 45 min. The HT22 cells were then washed three times in PBS and the fluorescent intensity of DHE was measured at 360/460 nm (*ex*/*em*) using a SPARK 10M fluorescent microplate reader (Tecan, Männedorf, Switzerland). Fluorescent images were taken using an IX50 fluorescent microscope equipped with a CCD camera (Olympus, Tokyo, Japan).

### 2.7. Measurement of Mitochondrial Membrane Potential

Cells were treated with 5 mM glutamate with or without SSF2-2 and NAC for 8 h and then 100 nM Tetramethylrhodamine methyl ester (TMRM; Thermo Fisher Scientific, Waltham, MA, USA), a cationic potentiometric fluorescent dye, was added to each well (1 × 10^4^ cells per well) containing adherent and non-adherent cells and incubated for another 30 min. The HT22 cells were then washed three times in PBS and the fluorescent intensity of TMRM was measured at 514/610 nm (*ex*/*em*) using a SPARK 10M fluorescent microplate reader (Tecan, Männedorf, Switzerland). Fluorescent images were taken using an IX50 fluorescent microscope equipped with a CCD camera (Olympus, Tokyo, Japan).

### 2.8. Measurement of Apoptotic Cell Death 

Apoptotic cells were quantified following annexin V/propidium iodide (PI) or TUNEL. Cells were treated with 5 mM glutamate with or without compounds (1–3) for 10 h and then harvested. These cells were washed with PBS, and then stained with annexin V Alexa Fluor 488 (Invitrogen, Eugene, OR, USA) in annexin binding buffer for 20 min, followed by staining with PI in annexin binding buffer for 2 min in accordance with the manufacturer’s instructions. Fluorescent images were collected using a Tali image-based cytometer. The percentage of apoptotic cells was calculated using the TaliPCApp (version 1.0). TUNEL assay was performed using TUNEL apoptosis detection kit (Abbkine Scientific Co., Ltd., CA, USA) according to the manufacturer’s instructions. The HT22 cells were then washed three times in PBS and the fluorescent intensity of TUNEL was measured at 490/520 nm (*ex*/*em*) using a SPARK 10M fluorescent microplate reader (Tecan, Männedorf, Switzerland). Fluorescent images were taken using an IX50 fluorescent microscope equipped with a CCD camera (Olympus, Tokyo, Japan).

### 2.9. Western Blot Analysis

Cells were treated with 5 mM glutamate with or without compounds (1–3) for 8 h and then harvested. These cells were lysed using RIPA buffer (Cell Signaling Technology, Inc., Danvers, MA, USA) containing 1X EDTA-free protease inhibitor cocktail (Roche, Indianapolis, IH, USA) and 1 mM phenylmethylsulfonyl fluoride (PMSF). Protein isolates (20 μg) were then separated by SDS-polyacrylamide gel electrophoresis and transferred to a polyvinylidene difluoride membrane (Merck Millipore, Darmstadt, Germany). These membranes were incubated with primary antibodies against Nrf2 (# 12721S, 1:1000), HO-1 (# 86806S, 1:1000), Cytochrome *c* (# 11940S, 1:1000), cleaved caspase-9 (# 70750S, 1:1000), cleaved caspase-3 (# 9661S, 1:1000), and glyceraldehyde 3-phosphate dehydrogenase (GAPDH) (# 2118S, 1:1000) for 1 h at room temperature, followed by incubation with secondary antibodies for 1 h at room temperature. All antibodies were purchased from Cell Signaling Technology. Immunoreactive bands were detected using SuperSignal West Femto Maximum Sensitivity Substrate (Thermo Scientific, Rockford, IL, USA) and quantified using ImageJ. 

### 2.10. Statistical Analysis

All assays were performed with triplicate samples and were repeated at least three times. All analyses were conducted using SPSS Statistics ver. 19.0 (SPSS Inc., Chicago, IL, USA). Nonparametric comparisons of samples were conducted using the Kruskal–Wallis test and a *p* value of <0.05 was considered statistically significant. 

## 3. Results

### 3.1. Effects of SSF2-1, SSF2-2, and SSF2-3 on Cell Viability after Glutamate Treatment

**Figure 1:**
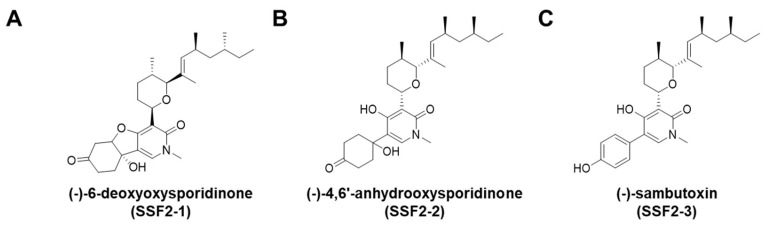
Structures of (**A**) SSF2-1, (**B**) SSF2-2, and (**C**) SSF2-3 isolated from cultures of *Fusarium lateritium* SSF2.

Fungal strain, SSF2, associated with *Cornus officinalis* fruits, was isolated and identified as *Fusarium lateritium* by ITS sequencing. Since the organic extracts of its cultures exhibited a unique UV spectroscopic pattern when subjected to HPLC-UV, the strain was selected for large-scale chemical analysis and cultivated in PDA media. A series of chromatographic separations isolated three tricyclic pyridone alkaloids which were identified as 6-deoxyoysporidinone (SSF2-1), 4,6′-anhydrooxysporidinone (SSF2-2), and sambutoxin (SSF2-3) by spectroscopic analysis (Figure 1). 

**Figure 2:**
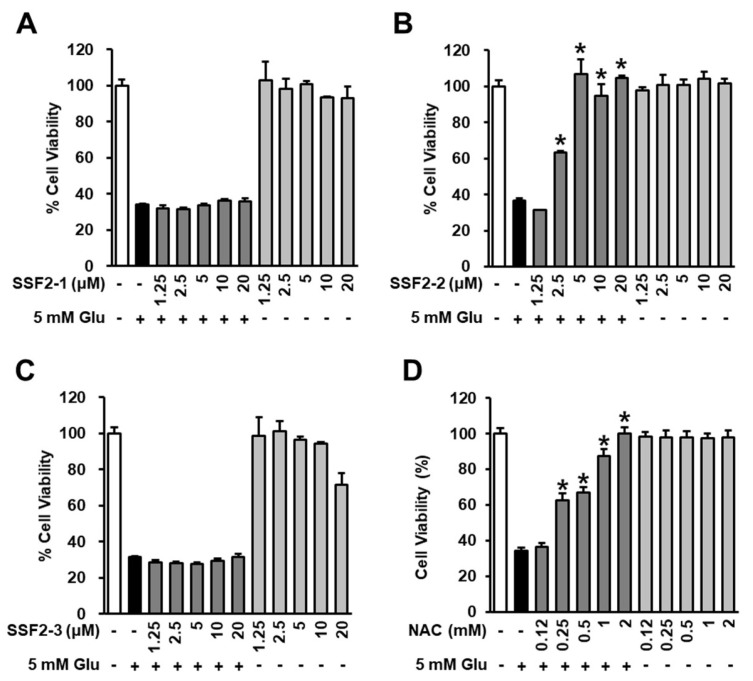
Effects of SSF2-1, SSF2-2, and SSF2-3 on cell viability after glutamate treatment. HT22 cells were incubated for 24 h with 5 mM glutamate (Glu) with or without (**A**) SSF2-1, (**B**) SSF2-2, (**C**) SSF2-3, and (**D**) N-acetylcysteine (NAC) at various concentrations. Cell viability was assessed using the Ez-CyTox reagent. Data represent the mean ± S.E.M., n = 3, * *p* < 0.05 compared with the glutamate-treated control.

MTT assay was used to evaluate the neuroprotective effect of SSF2-1, SSF2-2, SSF2-3, and NAC on HT22 cells treated with 5mM glutamate at concentrations ranging from 1.25 to 20 μM. When HT22 cells were treated with SSF2-1, SSF2-2, and NAC alone, there was no toxicity, but SSF2-3 was toxic at 25 μM. After treatment with 25 μM SSF2-3, cell viability was decreased by 69.2%. MTT assay results indicated that 5 mM glutamate deceased HT22 cell viability by 36%, and 6-deoxyoysporidinone (SSF2-1) exerted no protective effect on deceased HT22 cell viability after exposure to glutamate (Figure 2A). However, co-treatment with 4,6′-anhydrooxysporidinone (SSF2-2) decreased glutamate-induced cell death, with HT22 viability increasing by 63.1%, 106.5%, 95.2%, and 104.8% when HT22 cells were treated with 2.5, 5, 10, and 20 μM 4,6′-anhydrooxysporidinone, respectively compared to the glutamate control (Figure 2B). Sambutoxin (SSF2-3) also had no protective effect on cell viability after glutamate treatment (Figure 2C). Interestingly, SSF2-2 was more effective than NAC. At a concentration of 1 mM of NAC, LLC-PK1 cell viability was 87.3 ± 4.1%, and at 10mM it reached 99.9 ± 3.8%. 

### 3.2. Inhibitory Effects of SSF2-2 on Glutamate-Induced ROS Generation

**Figure 3:**
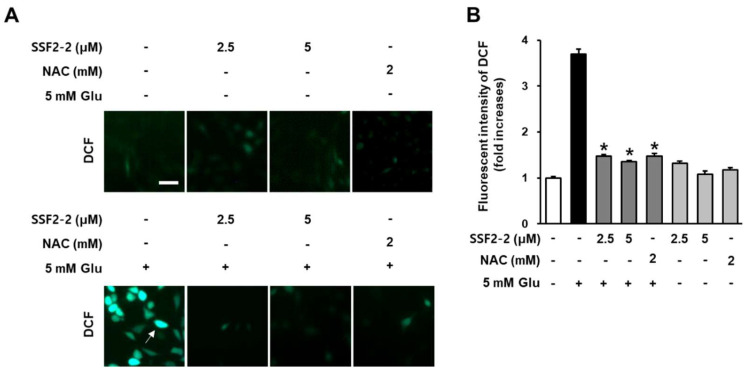
Effects of SSF2-2 on glutamate-induced increases in intracellular reactive oxygen species (ROS). HT22 cells were incubated for 24 h with 5 mM glutamate (Glu) with or without SSF2-2 and N-acetylcysteine (NAC), and then stained with DCFDA. (**A**) Fluorescent images were collected using a fluorescent microscope; (**B**) Fold increases in fluorescent intensity of DCF. White scale bar, 40 μm. Data represent the mean ± S.E.M., n = 3, * *p* < 0.05 compared with the glutamate-treated group.

We determined the antioxidant effect of 4,6′-anhydrooxysporidinone against glutamate-induced oxidative stress in HT22 cells using DCFH-DA, which is converted to DCF by ROS. When HT22 cells were treated with SSF2-2 and NAC alone, there was no change in the fluorescent dichlorofluorescein (green) compared to the control group. In the presence of ROS generated by glutamate, fluorescent dichlorofluorescein (green) was increased but it was significantly attenuated when HT22 cells were treated with 4,6′-anhydrooxysporidinone and NAC (Figure 3A). The fluorescence intensity of DCF can be used as an indicator of ROS activity. These results indicate that 5 mM glutamate increased the fluorescence intensity of DCF by 3.7-fold while co-treatment with 2.5 and 5 μM 4,6′-anhydrooxysporidinone reduced the DCF fluorescence intensity by 1.4- and 1.3-fold, respectively. Co-treatment with 2 mM NAC reduced the DCF fluorescence intensity by 1.4-fold (Figure 3B). 

**Figure 4:**
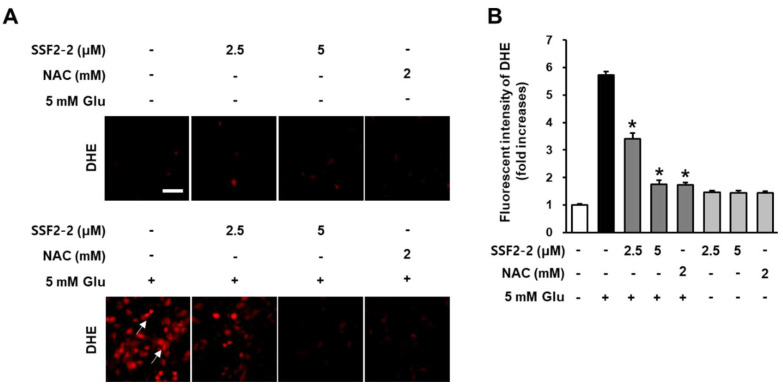
Effects of SSF2-2 on glutamate-induced increases in superoxide anion production. HT22 cells were incubated for 24 h with 5 mM glutamate (Glu) with or without SSF2-2 and N-acetylcysteine (NAC), and then stained with dihydroethidine (DHE). (**A**) Fluorescent images were collected using a fluorescent microscope; (**B**) Fold increases in fluorescent intensity of DHE. White scale bar, 40 μm. Data represent the mean ± S.E.M., n = 3, * *p* < 0.05 compared with the glutamate-treated group.

In addition, we determined the inhibitory effect of 4,6′-anhydrooxysporidinone against glutamate-induced increases in superoxide anion production in HT22 cells using DHE. The fluorescence intensity of DHE can be used as an indicator of superoxide productions. When HT22 cells were treated with SSF2-2 and NAC alone, there was no change in the DHE fluorescent (red) compared to the control group. After treatment with 5 mM glutamate, DHE fluorescent (red) increased but was significantly attenuated when HT22 cells were treated with 4,6′-anhydrooxysporidinone and NAC (Figure 4A). These results indicate that 5 mM glutamate increased the fluorescence intensity of DHE by 5.7-fold while co-treatment with 2.5 and 5 μM 4,6′-anhydrooxysporidinone reduced DHE fluorescence intensity by 3.4- and 1.7-fold, respectively. Co-treatment with 2 mM NAC reduced DHE fluorescence intensity by 1.7-fold (Figure 4B). 

### 3.3. Inhibitory Effects of SSF2-2 on Glutamate-induced Excessive Levels of Ca^2+^

**Figure 5:**
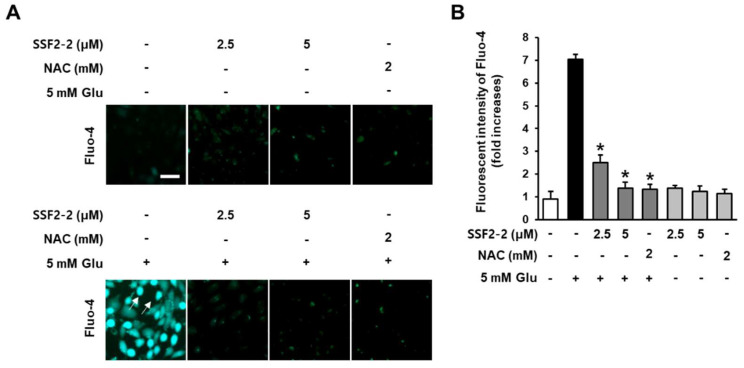
Effects of SSF2-2 on glutamate-induced excessive levels of Ca^2+^. HT22 cells were incubated for 24 h with 5 mM glutamate (Glu) with or without compound SSF2-2 and N-acetylcysteine (NAC), and then stained with Fluo-4 AM. (**A**) Fluorescent images collected using a fluorescent microscope. (**B**) Fold increases in fluorescent intensity of Fluo-4 AM. White scale bar, 40 μm. Data represent the mean ± S.E.M., n = 3, * *p* < 0.05 compared with the glutamate-treated group.

We evaluated the effect of 4,6′-anhydrooxysporidinone on levels of Ca^2+^ in glutamate-treated HT22 cells using a Fluo-4 AM dye. The fluorescence intensity of Fluo-4 AM with Ca^2+^ binding affinity was used as to quantify levels of intracellular Ca^2+^. As demonstrated by the microscopic images, when HT22 cells were treated with SSF2-2 and NAC alone, there was no change in the fluorescent intensity of Fluo-4 (green) compared to the control group. However, fluorescent intensity of Fluo-4 (green) was increased after treatment with 5 mM glutamate, but it was significantly attenuated when HT22 cells were co-treated with 4,6′-anhydrooxysporidinone (Figure 5A). The results indicated that 5 mM glutamate increased the fluorescence intensity of Fluo-4 AM by 7.1-fold. Co-treatment with 2.5 and 5 μM 4,6′-anhydrooxysporidinone reduced the fluorescence intensity of Fluo-4 AM by 2.5- and 1.3-fold, respectively. Co-treatment with 2 mM NAC reduced the fluorescence intensity of Fluo-4 AM by 1.3-fold (Figure 5B). 

### 3.4. The inhibitory Effects of SSF2-2 on Glutamate-induced Depolarization of Mitochondrial Membrane Potential

**Figure 6:**
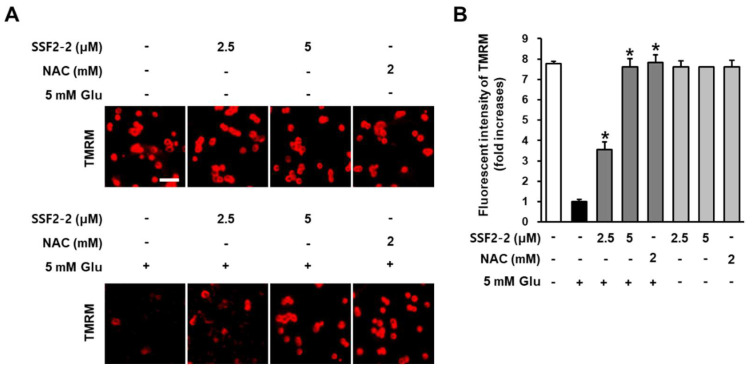
Effects of SSF2-2 on glutamate-induced depolarization of mitochondrial membrane potential. HT22 cells were incubated for 24 h with 5 mM glutamate (Glu) with or without compound SSF2-2 and N-acetylcysteine (NAC), and then stained with tetramethylrhodamine methyl ester (TMRM). (**A**) Fluorescent images collected using a fluorescent microscope. (**B**) Fold increases in fluorescent intensity of TMRM. White scale bar, 40 μm. Data represent the mean ± S.E.M., n = 3, * *p* < 0.05 compared with the glutamate-treated group.

Inhibitory effects of SSF2-2 on glutamate-induced depolarization of mitochondrial membrane potential was determined using potentiometric fluorescent dye TMRM that integrates into the mitochondria in a membrane potentially dependent manner [18]. When HT22 cells were treated with SSF2-2 and NAC alone, there was no change in the fluorescent intensity of TMRM (red) compared to the control group. After treatment with 5 mM glutamate, fluorescent intensity of TMRM (red) was decreased but it was significantly increased when HT22 cells were treated with 4,6′-anhydrooxysporidinone and NAC (Figure 6A). These results indicate that 5 mM glutamate decreased the fluorescence intensity of TMRM by 1.0-fold while co-treatment with 2.5 and 5 μM 4,6′-anhydrooxysporidinone increased TMRM fluorescence intensity by 3.5- and 7.6-fold, respectively. Co-treatment with 2 mM NAC increased TMRM fluorescence intensity by 7.8-fold (Figure 6B).

### 3.5. The Inhibitory Effects of SSF2-2 on Glutamate-Induced Apoptosis

**Figure 7:**
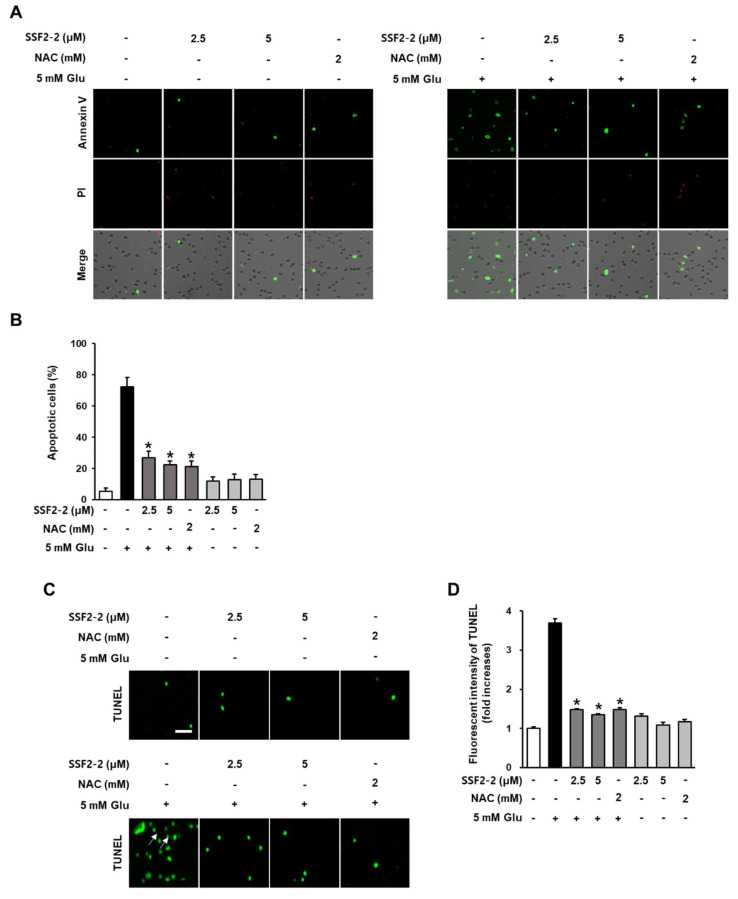
Effects of SSF2-2 on glutamate-induced apoptotic cell death. HT22 cells were incubated for 24 h with 5 mM glutamate (Glu) with or without compound SSF2-2 and N-acetylcysteine (NAC), and then stained with annexin V/propidium iodide (PI) or TUNEL. (**A**) Fluorescent images were collected using a Tali image-based cytometer after staining with annexin V/PI. (**B**) % of apoptotic cells were calculated using TaliPCApp (version 1.0). (**C**) Fluorescent images collected using a fluorescent microscope after staining with TUNEL. (**D**) Fold increases in fluorescent intensity of TUNEL. White scale bar, 40 μm. Data represent the mean ± S.E.M., n = 3, * *p* < 0.05 compared with the glutamate-treated group.

We determined the anti-apoptotic effect of 4,6′-anhydrooxysporidinone on glutamate treated HT22 cells using annexin V Alexa Fluor 488/PI staining and TUNEL staining. When HT22 cells were treated with SSF2-2 and NAC alone, there was no change in the fluorescence intensity of the annexin V-positive cells, indicating apoptotic cells. However, in the presence of glutamate, the fluorescence intensity of the annexin V-positive cells was increased, but this was significantly attenuated when HT22 cells were co-treated with 4,6′-anhydrooxysporidinone (Figure 7A). The percentage of apoptotic cells was analyzed using TaliPCApp (version 1.0) and the results indicated that 5 mM glutamate increased the percentage of apoptotic cells by 72.3% but co-treatment with 2.5 or 5 μM 4,6′-anhydrooxysporidinone reduced the percentage of apoptotic cells to 27.1% and 22.3%, respectively. Co-treatment with 2 mM NAC reduced the percentage of apoptotic cells to 2.1% (Figure 7B). When HT22 cells were treated with SSF2-2 and NAC alone, there was no change in the fluorescent intensity of TUNEL (green), indicating nuclear DNA fragmentation, compared to the control group. However, in the presence of glutamate, the fluorescent intensity of TUNEL (green) was increased, but this was significantly attenuated when HT22 cells were co-treated with 4,6′-anhydrooxysporidinone (Figure 7C). These results indicate that 5 mM glutamate increased the fluorescence intensity of TUNEL by 6.6-fold while co-treatment with 2.5 and 5 μM 4,6′-anhydrooxysporidinone reduced TUNEL fluorescence intensity by 4.1- and 2.1-fold, respectively. Co-treatment with 2 mM NAC reduced TUNEL fluorescence intensity by 2.1-fold (Figure 7D). 

### 3.6. Effects of SSF2-2 on Glutamate-induced Reductions in the Nrf2 and HO-1.

**Figure 8:**
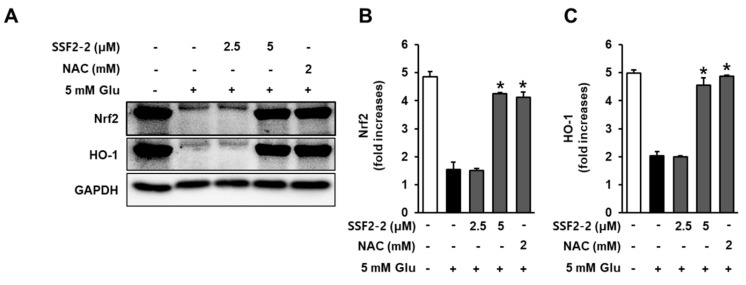
Effects of SSF2-2 on glutamate-induced reductions in the nuclear factor-erythroid-2–related factor 2 (Nrf2) and heme oxygenase-1 (HO-1). HT22 cells were incubated for 24 h with 5 mM glutamate (Glu) with or without compound SSF2-2 at 2.5 and 5 μM, and then subjected to Western blot. (**A**) Protein expression of Nrf2, HO-1, and GAPDH. Fold increases in protein expression of (**B**) Nrf2 and (**C**) HO-1. Data represent the mean ± S.E.M., n = 3, * *p* < 0.05 compared with the glutamate-treated group.

We evaluated the effect of 4,6′-anhydrooxysporidinone treatment on glutamate-induced reductions in the Nrf2 and HO-1 after 8 h of glutamate exposure. In the presence of glutamate, the Nrf2 and HO-1 decreased, but these reductions were significantly enhanced when HT22 cells were co-treated with 4,6′-anhydrooxysporidinone (Figure 8A). Quantitative data also showed that 5 mM glutamate resulted in reduction in the expression of Nrf2 by 1.5-fold, while co-treatment with 2.5, 5 μM 4,6′-anhydrooxysporidinone, and 2 mM NAC enhanced expressions of Nrf2 by 1.5-, 4.2-, and 4.1-fold, respectively (Figure 8B). Quantitative data also showed that 5 mM glutamate resulted in reduction in the expression of HO-1 by 2.1-fold while co-treatment with 2.5, 5 μM 4,6′-anhydrooxysporidinone, and 2 mM NAC enhanced expressions of HO-1 by 1.9-, 4.5-, and 4.8-fold, respectively (Figure 8C). 

### 3.7. Inhibitory Effects of SSF2-2 on Glutamate-Mediated Cytochrome c Release and Cleaved Caspase-9, -3

**Figure 9:**
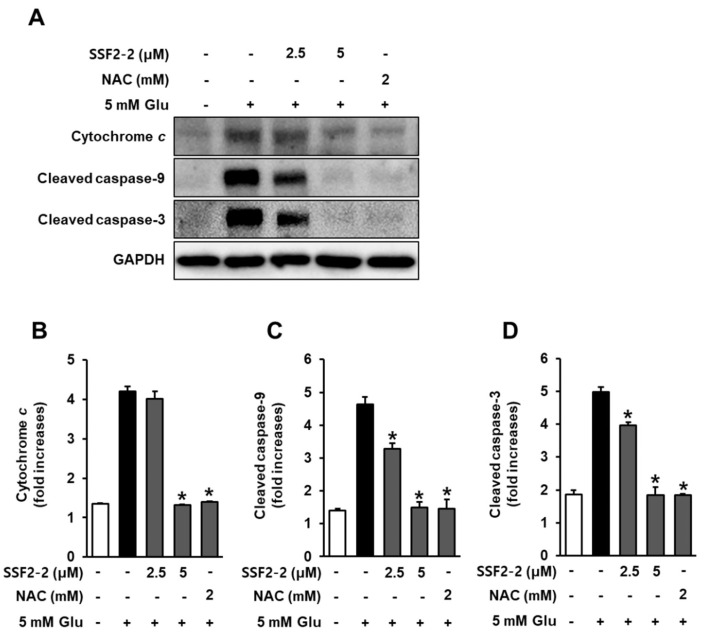
Effects of SSF2-2 on glutamate-induced cytochrome *c* release and cleaved caspase-9, -3. HT22 cells were incubated for 24 h with 5 mM glutamate (Glu) with or without compound SSF2-2 at 2.5 and 5 μM, and then subjected to western blot. (**A**) Protein expression of cytochrome *c*, cleaved caspase-9, cleaved caspase-3, and GAPDH. Fold increases in protein expression of (**B**) cytochrome *c*, (**C**) cleaved caspase-9, and (**D**) cleaved caspase-3. Data represent the mean ± S.E.M., n = 3, * *p* < 0.05 compared with the glutamate-treated group.

We evaluated the effect of 4,6′-anhydrooxysporidinone treatment on glutamate-induced cytochrome *c* release and cleaved caspase-9, -3 after 8 h of glutamate exposure. In the presence of glutamate, the expressions of cytochrome *c* and cleaved caspase-9, -3 increased, but these expressions were significantly attenuated when HT22 cells were treated with 4,6′-anhydrooxysporidinone (Figure 9A). Quantitative data also showed that 5 mM glutamate increased expression of cytochrome *c* by 4.2-fold, while co-treatment with 2.5, 5 μM 4,6′-anhydrooxysporidinone, and 2 mM NAC reduced expression of cytochrome *c* by 4.1-, 1.3-, and 1.3-fold, respectively (Figure 9B). Quantitative data also showed that 5 mM glutamate increased expression of cleaved caspase-9 by 4.6-fold, while co-treatment with 2.5, 5 μM 4,6′-anhydrooxysporidinone, and 2 mM NAC reduced expression of cleaved caspase-9 by 3.2-, 1.4-, and 1.4-fold, respectively (Figure 9C). Quantitative data also showed that 5 mM glutamate increased expression of cleaved caspase-3 by 4.9-fold, while co-treatment with 2.5, 5 μM 4,6′-anhydrooxysporidinone, and 2 mM NAC reduced expression of cleaved caspase-3 by 3.9-, 1.4-, and 1.4-fold, respectively (Figure 9D).

## 4. Discussion

The finding of the present study was that 4,6′-anhydrooxysporidinone isolated from *F. lateritium* exerts exhibited the protective effect on deceased HT22 cell viability after exposure to glutamate. Interestingly, 4,6′-anhydrooxysporidinone was more effective than NAC, which is a popular positive control as an antioxidant in the study on glutamate-induced cell death for its ability to minimize oxidative stress [19,20]. This was confirmed by evaluating the reduced intracellular ROS, accumulation of intracellular ROS, increases in superoxide anion production, Ca^2+^, depolarization of mitochondrial membrane potential, and apoptotic cell death. It is well known that the rapid ROS, including superoxide triggered by glutamate can lead to neuronal cell death [21], and apoptosis pathways [22]. Earlier studies reported that antioxidants exert neuroprotective effects by blocking oxidative stress and apoptosis in vitro and in vivo [23]. In the present study, in the presence of ROS generated by glutamate, fluorescent dichlorofluorescein was increased, but it was significantly attenuated when HT22 cells were co-treated with 4,6′-anhydrooxysporidinone. This decreased fluorescence intensity suggests that 4,6′-anhydrooxysporidinone can inhibit oxidative stress triggered by the presence of glutamate. In addition, we determined the inhibitory effect of 4,6′-anhydrooxysporidinone against glutamate-induced increases in superoxide anion production in HT22 cells. The fluorescence intensity of DHE can be used as an indicator of superoxide productions. After treatment with glutamate, DHE fluorescent was increased, but it was significantly attenuated when HT22 cells were co-treated with 4,6′-anhydrooxysporidinone. This decreased fluorescence intensity suggests that 4,6′-anhydrooxysporidinone can inhibit superoxide anion production triggered by the presence of glutamate. A number of studies have reported that glutamate depletes intracellular glutathione through the accumulation of ROS in HT-22 cells, resulting in toxicity and cell death [24,25,26]. The inhibitory effect of 4,6′-anhydrooxysporidinone on ROS generation and increases in superoxide anion production in HT22 cells may contribute to its protective effect against glutamate-induced HT22 cell death. 

It is well known that glutamate-induced HT22 cell death is by the excessive levels of Ca^2+^ [27]. Thus, we evaluated the effect of 4,6′-anhydrooxysporidinone on levels of Ca^2+^ in glutamate-treated HT22 cells using a Fluo-4 AM dye, a membrane permeable fluorescent indicator for Ca^2+^. The fluorescent intensity of Fluo-4 was increased after treatment with 5 mM glutamate, but it was significantly attenuated when HT22 cells were co-treated with 4,6′-anhydrooxysporidinone. This decreased fluorescence intensity suggests that 4,6′-anhydrooxysporidinone inhibits glutamate-induced excessive levels of Ca^2+^, resulting in HT22 cell death. The excessive levels of Ca^2+^ leads to dysfunction of mitochondrial through the depolarization of mitochondrial membrane [28].

Depolarization of mitochondrial membrane potential is also involved in glutamate-induced neuronal cell death [29]. Inhibitory effects of 4,6′-anhydrooxysporidinone on glutamate-induced depolarization of mitochondrial membrane potential was determined using potentiometric fluorescent dye TMRM. In the present study, after treatment with 5 mM glutamate, fluorescent intensity of TMRM was decreased, this decreased fluorescence intensity suggests that glutamate inhibited accumulation of TMRM in active mitochondria with intact membrane potentials. However, the fluorescent intensity of TMRM was significantly increased when HT22 cells were treated with 4,6′-anhydrooxysporidinone. This increased fluorescence intensity suggests that 4,6′-anhydrooxysporidinone can inhibit depolarization of mitochondrial membrane potential triggered by the presence of glutamate. It may be also associated with the inhibitory effects of 4,6′-anhydrooxysporidinone on glutamate-induced ROS generation. Previous studies have shown that the depolarization of mitochondrial membrane potential is associated with ROS production in glutamate-induced neuronal cell death [18].

Oxidative stress can result in apoptotic cell death in the presence of high concentrations of glutamate [30,31]. Therefore, we determined the anti-apoptotic effect of 4,6′-anhydrooxysporidinone on glutamate treated HT22 cells using annexin V Alexa Fluor 488/PI staining and TUNEL staining. In the presence of glutamate, the fluorescence intensity of the annexin V-positive cells was increased, but this was significantly attenuated when HT22 cells were co-treated with 4,6′-anhydrooxysporidinone. In addition, in the presence of glutamate, the fluorescent intensity of TUNEL indicating nuclear DNA fragmentation, was increased, but this was significantly attenuated when HT22 cells were co-treated with 4,6′-anhydrooxysporidinone. These decreases following 4,6′-anhydrooxysporidinone treatment indicates that this compound inhibits apoptotic cell death in the presence of glutamate. 

The transcription factor Nrf2 and activated HO-1 through Nrf2 transactivation enhance activities of antioxidant enzyme such as SOD and CAT, resulting in the enhancement of cellular antioxidant function, and inhibits the depolarization of mitochondrial membrane [32]. In the present study, in the presence of glutamate, the Nrf2 and HO-1 decreased, but these reductions were significantly enhanced when HT22 cells were co-treated with 4,6′-anhydrooxysporidinone, which may be involved in the protective effect of 4,6′-anhydrooxysporidinone against toxicity in glutamate-treated HT22 cells. 

In response to increased ROS and depolarization of mitochondrial membrane, cytochrome *c* located in the mitochondrial intermembrane/intercristae spaces is released. It is a pro-apoptotic cytokines that combines with the apoptotic protease activating factor 1 (Apaf-1), sequentially recruits caspase-9. An executioner caspase-3 in mitochondria-mediated apoptosis is activated by activation of caspase-9 in glutamate-treated HT22 cells [32]. In the present study, in the presence of glutamate, the expressions of cytochrome *c* and cleaved caspase-9, -3 increased, but these expressions were significantly attenuated when HT22 cells were treated with 4,6′-anhydrooxysporidinone. These decreases following 4,6′-anhydrooxysporidinone treatment indicate that this compound inhibits apoptotic cell death through the downregulation of cytochrome *c* release and cleaved caspase-9, -3 in glutamate-treated HT22 cells. Taken together with the previous results 4,6′-anhydrooxysporidinone exerts a protective effect against glutamate-induced apoptotic cell death in HT22 cells by inhibiting ROS overproduction, reducing elevated intracellular Ca^2+^ levels and inhibiting depolarization of mitochondrial membrane potential. At least partly, this role of 4,6′-anhydrooxysporidinone in glutamate-treated HT22 cells is associated with the enhancement of Nrf2/HO-1 and inhibition of cytochrome *c* and cleaved caspase-9, -3.

## 5. Conclusions

In this study, we demonstrated the protective effect of 4,6′-anhydrooxysporidinone isolated from the endophytic fungus *Fusarium lateritium* SSF2 against glutamate-induced HT22 cell death. 4,6′- Anhydrooxysporidinone protected HT22 cells from glutamate-induced cytotoxicity, accumulation of intracellular ROS, increases in superoxide anion production, intracellular Ca^2+^ levels, depolarization of mitochondrial membrane potential, and apoptotic cell death. In addition, 4,6′- Anhydrooxysporidinone inhibited glutamate-induced HT22 cell death related to the accumulation of intracellular ROS, possibly in part through activation of glutamate-mediated Nrf2/HO-1 pathways. 4,6′- Anhydrooxysporidinone also inhibited apoptotic cell death through the inhibition of cytochrome *c* and cleaved caspase-9, -3. Our results provided evidence for the neuroprotective properties of 4,6′-anhydrooxysporidinone and further support the profiling of natural products from fungi for application in the treatment of neurodegenerative diseases.
